# Fetal cardiac rhabdomyomas susceptible to prenatal treatment with mTOR inhibitors: literature review and proposal of a prenatal management algorithm

**DOI:** 10.3389/fmed.2025.1711774

**Published:** 2025-12-08

**Authors:** Alfonso Martinez-Garcia, Omar A. Tirado-Aguilar, Sandra Acevedo-Gallegos, Juan M. Gallardo-Gaona, Berenice Velazquez-Torres, Jose A. Ramirez-Calvo, Dulce M. A. Camarena-Cabrera, Maria J. Rodriguez-Sibaja, Mario I. Lumbreras-Marquez, Yazmin Copado-Mendoza

**Affiliations:** 1 Maternal Fetal Medicine Department, Instituto Nacional de Perinatología Isidro Espinosa de los Reyes, Mexico City, Mexico; 2 Fetal Cardiology Clinic, Instituto Nacional de Perinatología Isidro Espinosa de los Reyes, Mexico City, Mexico; 3 Bioinformatics and Biostatistics Department, Instituto Nacional de Perinatología Isidro Espinosa de los Reyes, Mexico City, Mexico; 4 Epidemiology and Public Health Department, Universidad Panamericana School of Medicine, Mexico City, Mexico

**Keywords:** fetal cardiac rhabdomyomas, mTOR inhibitors, sirolimus, everolimus, prenatal therapy, transplacental treatment, echocardiographic criteria

## Abstract

Certain types of fetal cardiac rhabdomyomas can lead to severe complications, including intrauterine death, yet no specific criteria have been established for the prenatal use of pharmacological therapies to mitigate the impact of rhabdomyomas. We conducted a narrative review of case reports and case series published between January 1, 2000, and February 28, 2025, identified through PubMed, Scopus, Web of Science, and Google Scholar, describing the prenatal use of mammalian target of rapamycin inhibitors in this context. Thirteen studies reporting on 15 fetuses were included. Five fetuses (33.3%) had a single rhabdomyoma, and 10 (66.6%) had multiple lesions. Prenatal genetic testing for Tuberous Sclerosis Complex was performed in 9 cases (60%): 1 with a TSC1 mutation, 7 with TSC2 mutations, and 1 negative. Sirolimus was the most frequently used inhibitor (86.6%), while everolimus was used in 2 cases (13.3%). The main indication for treatment was progressive tumor growth causing outflow obstruction and/or hemodynamic compromise, including reduced cardiac output, arrhythmias, and fetal hydrops. Therapy was initiated at a median of 30.0 weeks (IQR 26.7–33.1) and completed at 38.0 weeks (IQR 36–39). All reports documented tumor reduction and improved cardiac function, though regrowth occurred in 5 cases (33.3%) after discontinuation. No fetal or neonatal deaths were reported, and none required postnatal cardiac surgery before discharge. Based on these findings, we proposed echocardiographic criteria to identify suitable candidates, including inflow/outflow tract obstruction, severe atrioventricular valve insufficiency, tachyarrhythmia, impaired cardiac function, or hydrops, and developed a structured prenatal management algorithm. Prenatal therapy with mTOR inhibitors, therefore, appears to improve fetal cardiac function by reducing tumor burden and may contribute to better perinatal outcomes, although validation in future studies is required. TSC1: urn:lsid:hgnc.org: HGNC:12362

TSC2: urn:lsid:hgnc.org: HGNC:12363 Sirolimus: urn:lsid:ebi.ac.uk:chebi:9168 Everolimus: urn:lsid:ebi.ac.uk:chebi:68478.

## Introduction

Fetal cardiac rhabdomyomas (FCR) are the most commonly diagnosed prenatal cardiac tumors (1), representing 60–86% of primary fetal cardiac tumors (2), with an incidence of 0.12% on fetal echocardiography (3). FCR are typically diagnosed between 20 and 30 weeks of gestation, during anatomy ultrasound screening, with some reports of diagnoses as early as 17 weeks of gestation (4). Fetal echocardiography is crucial in detecting complications caused by these tumors, such as cardiomegaly, pericardial effusion, arrhythmias, ventricular outflow obstruction, and hypokinesia (5).

Most FCRs have a benign clinical course and usually involute spontaneously after birth; however, when they are large, numerous, or located in specific anatomical sites, they can cause serious complications such as arrhythmias, valvular abnormalities, ventricular outflow tract obstruction, heart failure, pericardial effusion, hydrops, and even fetal death (6). Because 13% regress spontaneously at birth and 58% at postnatal follow-up (2), the current recommendation is often to take a conservative approach, especially for younger patients and smaller tumors, to avoid the risks associated with surgery (1). Postnatally, until recently, surgery and medical treatment with antiarrhythmic drugs were the only options for FCR, but these can cause hemodynamic issues. However, surgical removal is associated with significant risks and is not feasible if the tumors are multifocal, infiltrative, or very large (7, 8). In these cases, the use of mTOR (mammalian target of Rapamycin) inhibitor therapy has shown benefits (9). The mTOR inhibitors (mTORi) are a class of drugs initially developed as antifungals, and later found to possess immunosuppressive and antiproliferative properties (10). Consequently, they have been used to treat conditions such as tuberous sclerosis complex (TSC), lymphangioleiomyomatosis (11), psoriasis, and malignant tumors (12), as well as in renal and cardiac transplant recipients (13, 14). The prototype mTORi is sirolimus (SRL; rapamycin, Rapamune®), a lipophilic macrocyclic lactone produced by a strain of *Streptomyces hygroscopicus*, first isolated from a soil sample collected on Rapa Nui (also known as Easter Island) (15). The pharmaceutical forms of Rapamune® are 0.5 mg, 1 mg, and 2 mg (16). Everolimus (EVR; RAD100, Certican®) is a derivative of SRL, obtained through O-alkylation at position 40 (17). The pharmaceutical forms of everolimus differ by brand, Certican®—used for solid organ transplant rejection prophylaxis—is available as 0.25 mg, 0.5 mg, and 0.75 mg tablets, whereas Afinitor®—used in oncology—is available as 2.5 mg, 5 mg, 7.5 mg, and 10 mg tablets (18). Both SRL and EVR inhibit the mTOR pathway, causing cell cycle arrest in the early G1-S phase, and reducing the manifestations of TSC due to their antitumor activity (15, 17).

Postnatal treatment with mTORi in children with cardiac rhabdomyomas (CR) that cause hemodynamic problems and are linked to TSC has achieved a clinical improvement in 90.9%, with a reduction in tumor size in 95.1%. Mild side effects such as dyslipidemia, transient lymphopenia, mouth ulcers, changes in phosphate levels, diarrhea, and constipation were observed. This is why, currently, postnatal use of mTORi can be considered a temporary and safe treatment for CR in children with TSC (19, 20).

In recent years, mTOR inhibitors have been introduced as a new prenatal therapeutic option for FCR that causes severe complications. The first case of successful birth in a liver-transplant woman who received SRL during the first 6 weeks of gestation was described in 2004 by Jankowska et al. (21). In 2011, Framarino-dei-Malatesta et al. (22) described a case of pregnancy in a renal-transplant patient who received SRL treatment throughout gestation. Regarding EVR, the first case of successful pregnancy in a female kidney transplant recipient exposed to EVR throughout gestation was described by Veroux et al. (23) in 2011. All these cases demonstrated that the use of SRL or EVR might not be an absolute contraindication during pregnancy (15).

Tiberio et al. (24) were the first to report that EVR treatment given to a patient with subependymal giant cell astrocytoma resulted in regression of cardiac rhabdomyomas. Subsequently, Aw et al. (25) reported that the regression of cardiac rhabdomyomas treated with everolimus is 11.8 times faster than natural regression. In 2018, Barnes et al. (26) documented the use of maternal SRL therapy for fetal cardiac rhabdomyomas causing cardiac outflow tract obstruction and supraventricular tachycardia. After sirolimus administration, in utero tumor regression was observed, allowing for the pregnancy to continue until 36 weeks of gestation. However, after birth, the tumor continued to grow as long as the drug was not administered, leading to the indication of SRL in the infant at 2 months of age, with a target level of 10–15 ng/mL. After 3 weeks, tumor regression was again evident. This evidence suggests that prenatal use of mTORi may be possible in selected cases, especially those with FCR causing severe complications, demonstrating rapid tumor regression and improved fetal hemodynamics. Despite this, the available evidence primarily consists of isolated case reports or small series, and its use during pregnancy appears to be safe (2). However, this practice lacks clear clinical guidelines regarding indications, dosing, monitoring, and long-term maternal and fetal outcomes. Currently, no consensus, guidelines, or clinical trials have established definitive protocols.

The main aim of this study was to conduct a narrative review of the international literature on reports and case series of fetuses with CR that received prenatal treatment with any mTORi. As specific objectives, echocardiographic criteria are proposed to classify fetuses diagnosed with CR as candidates for prenatal treatment with mTORi. Additionally, a management algorithm has been developed to provide guidelines for the use of mTORi in FCR, based on the most current evidence from the international literature.

## Methods

### Methodology of the narrative review of the literature

A narrative review of the scientific literature on the prenatal use of mTOR inhibitors in FCR was conducted to synthesize and contextualize the available findings from case reports. This approach was selected due to the limited number of controlled studies and the novel nature of the topic.

The search was conducted in the electronic databases PubMed, Scopus, Web of Science, and Google Scholar. To improve the search sensitivity and ensure the inclusion of relevant studies, the search terms were customized to fit each database’s format, using Boolean operators to combine synonyms and related terms. In PubMed, MeSH terms were used; in Scopus and Web of Science, structured operators were employed; and in Google Scholar, a free-text search was performed with key phrases. Terms included keyword combinations such as “fetal rhabdomyoma,” “cardiac rhabdomyoma,” “mTOR inhibitors,” “sirolimus,” “everolimus,” “prenatal,” and “transplacental therapy.” The search was limited to publications from January 1, 2000, to February 28, 2025, involving humans and available in English or Spanish.

Priority was given to including relevant literature without applying strict selection criteria typical of other review types (e.g., systematic reviews). Case reports and case series were eligible if they documented prenatal diagnosis of FCR by fetal echocardiography and included key variables such as indication, type and dose of mTORi used, gestational age at administration, reported maternal and fetal adverse effects, description of FCR (number of tumors and size of the largest), prenatal genetic study of TSC, and clinical cardiac evolution during follow-up. We did not exclude articles based on methodological quality if the content was relevant to the study objectives. Conference abstracts were not included. Supplementary Table 1 lists the search terms used, along with the number of initial and final articles retrieved from each database. Supplementary Table 2 presents the articles selected from each database.

### Statistical analysis

Two reviewers independently extracted data. Any disagreements were resolved through consensus. This process helped improve reliability in analyzing information from the included studies. The data was collected and analyzed using the REDCap (RRID:SCR_003445) (Research Electronic Data Capture) platform. Continuous variables were described using measures of central tendency and dispersion [mean (standard deviation) and median [interquartile range (IQR)]] calculated directly with REDCap’s integrated statistical functions. Categorical variables were presented as absolute numbers and percentages. Inferential statistical methods and heterogeneity analysis were not performed.

### Development of the management algorithm

After compiling information from international reports and case series on FCR treated with mTORi, along with documented experiences of their long-term use in transplant patients, a management and follow-up algorithm was developed for fetuses diagnosed with CR that are candidates for mTORi treatment. This algorithm also includes guidelines for maternal and fetal monitoring, as well as potential side effects of the therapy.

### Ethical considerations

Since this was a literature review of previously published and anonymized data, this study is exempt from Ethics Committee approval. The principles of the Declaration of Helsinki were followed, and REDCap was used as the data management platform, which complies with international confidentiality and quality standards, including the Health Insurance Portability and Accountability Act (HIPAA) of the United States.

## Results

### Narrative literature review

We identified 13 studies, which consisted of case reports and case series, providing information on 15 fetuses with CR who received prenatal treatment with mTORi. These studies were organized by year of publication (6, 26–37). Table 1 outlines the characteristics of the 15 fetuses with CR that received mTORi, of whom 5 had single tumors (33.3%) and 10 had multiple tumors (66.6%). Prenatal genetic testing for TSC was performed in 9 fetuses (60%), with one fetus reporting the TSC 1 gene (6.6%), 7 fetuses with the TSC 2 gene (46.6%), and 1 fetus with a negative result for TSC (6.6%).

**Table 1:** Prenatal treatment with mTORi for fetal cardiac rhabdomyomas in 15 fetuses (13 studies), guidelines used and postnatal evolution.

Author/publication year	mTORi indication	Prenatal genetic testing TSC1/TSC2	No. FCRs/largest FCR (mm) at start mTORi/location	mTORi type/ dose	Maternal mTORi target level (ng/ml)	GA mTORi initiation (weeks)	GA mTORi termination (weeks)	mTORi levels at birth: cord blood/maternal (ng/ml)	GA delivery (weeks)/mode of delivery	Maternal S. E	FGR detected	Prenatal reduction in tumor size	Cardiac tumor outcomes after delivery
Barnes et al. (26)/2018	Bilateral outflow tract obstruction, SVT, impending fetal hydrops	TSC 1	Multiple/12.6 × 20.7 mm/ LV	SRL/12 mg/day (6.3 mg/m^2^) during first 48 h, additional 22 mg/day (11.7 mg/m2); then 13 ± 2 mg/day (6.8 ± 1.04 mg/m^2^/day)	10–15	30	36.3	11.3/6.9	36.3/C-section	No	No	Yes	After birth, the tumor progressed while the infant was not receiving therapy. Sirolimus was initiated at 2 months of age, and after 3 weeks, tumor regression was observed again
Vachon-Marceau et al. (27)/2019	Ongoing growth with pericardial effusion and deterioration of cardiac function	TSC 2	Multiple/47 × 39 mm/LV	SRL/15 mg/day loading dose; then 5–8 mg/day	10–15	31.4	36.0	NA	39/NA	No	No	Yes	Sirolimus was discontinued at 36 weeks of gestation, followed by an increase in the size of the rhabdomyoma. The postnatal echocardiogram confirmed multiple cardiac tumors, with mildly reduced
Park et al. (28)/2019	Maternal TSC with pulmonary lymphangioleimyomatosis and fetal cardiac rhabdomyoma	TSC 2	Multiple/12 × 9 mm/LV	SRL/4 mg/day loading dose (2.2 ng/mL serum level); then 12 mg/day	10–15	23	39	33.2/25.0	39/NA	No	No	Yes	At 29.5 weeks of gestation, no cardiac tumor was observed. One day after birth, echocardiography showed no intracardiac tumor.
Pluym et al. (6)/2020	Ongoing growth, pericardial effusion, decreased ejection fraction, and mitral regurgitation	TSC 2	Multiple/45 × 35 mm/Apex	SRL/10 mg/day loading dose; then 6–10 mg/day	10–15	28	35	3.3 /3.4	36.6/Vaginal	Preeclampsia	Yes	Yes	The postnatal echocardiogram demonstrated a large cardiac rhabdomyoma without functional flow obstruction. At 6 months of age, stable cardiac rhabdomyomas were observed, and sirolimus reinitiation was not required.
Cavalheiro et al. (29)/2021	Ongoing growth of cardiac and brain lesions	NA	Multiple/10 × 7 mm /IVS	EVR/ 10 mg/day loading dose (8.4 ng/mL serum level)	3–15	NA	39	NA	39/C-section	No	No	Yes	The postnatal echocardiogram revealed a small atrial lesion. At 4 days of life, the patient resumed treatment with everolimus. At 36 months of age, neuropsychomotor development was normal. The patient’s EEG was normal, and no seizures were ever reported.
Dagge et al. (30)/2021	Ongoing growth with fetal arrhythmia and mild tricuspid regurgitation	NA	Single/6 × 6 mm/Septal leaflet TV	SRL/4 mg/day loading dose, then 10 mg/day (13.8 ng/mL serum level)	10–15	26	39	NA /16.7	39/C-section	NA	No	Yes	The first postnatal evaluation showed sinus rhythm and normal cardiac function.
Ebrahimi-Fakhari et al. (31)/2021	Patient 1 ongoing growth and worsening LVOT obstruction	TSC 2	Multiple/NE/NE	SRL/1 mg/day for 2 days, then 3 mg/day	5–15	35.2	39.1	NA /NA	39.1/NA	No	No	Yes	Reduction in the size of rhabdomyomas and resolution of LVOT obstruction. Birth without evidence of cardiac or respiratory compromise.
	Patient 2 Giant tumor encapsulating the left ventricle	TSC 2	Multiple/39 × 34 × 28 mm/ LV + apex	SRL/3 mg/day	NA	33.1	36.6	NA /NA	36.6/NA	No	No	Yes	Mass shrank gradually, no obstruction of the LVOT detected
	Patient 3 ongoing growth and worsening LVOT obstruction	TSC 2	Multiple/NA/NA	SRL/4 mg AM and 2 mg PM (~3.3 mg/m^2^/day)	9.5	34.0	38.6	NA /NA	38.6/NA	No	No	Yes	An echocardiogram performed on the first day of life demonstrated multiple rhabdomyomas. The right and left ventricles were of normal size and normal systolic function
Will et al. (32)/2023	Giant rhabdomyoma, tricuspid regurgitation, and pericardial effusion	NA	Single/21.5 × 39.7 mm/RA with extension to RV/LV	SRL/4 mg/day (2.16 mg/m^2^/day serum level)	9–12	27	38	1.59 / 1.2	39.1/Vaginal	Aphthous ulcer	No	Yes	Postnatal echocardiography showed only partial coverage of the tricuspid valve without inflow obstruction. Additional treatment with everolimus was started and, after eight more weeks, the tumor in the right atrium, as well as the rhabdomyoma of the posterior wall of the left ventricle, disappeared
McLoughlin et al. (33)/2023, Maász et al. (34)/2023	Giant rhabdomyoma, mitral and tricuspid regurgitation, and hydrops fetalis	NA	Multiple/44 × 44 mm/ IVS	SRL/2 mg/m^2^/day divided twice daily	10–12	30	36	NA /NA	36/C-section	NA	No	Yes	Inadequate cardiac output due to left ventricular systolic dysfunction. Epinephrine, milrinone, prostaglandins along with oral everolimus was maintained. At 3 months, the tumor measured 9 × 15 mm without LVOT obstruction
Maász et al. (34)/2023	Ongoing growth	TSC 2	Multiple/31 × 32 mm/LV	EVR/initial dose of 10 mg/day and adjusted to 5 mg/day from day 10	5–15	NA	NA	NA/NA	At term/C-section	NA	No	Yes	Postnatal cardiological examination revealed rhabdomyoma and mitral regurgitation
Griesman et al. (35)/2024	Ongoing growth, RVOT, and tricuspid regurgitation	Negative	Single/NA/IVS	SRL/NA	NA	23	34	NA/NA	37.6/Vaginal	Aphthous ulcer	NA	Yes	Postnatal echocardiography showed a larger tumor than that observed in the final fetal examination, but without significant hemodynamic alteration. At 2 months of age, the tumor increased in size and there was recurrence of ventricular outflow tract obstruction. Treatment with sirolimus was started for the next 4 months and the tumor almost disappeared.
Schenk et al. (36)/2024	Ongoing growth and pericardial effusion	NA	Single/Diameter of 49 mm/LV	SRL/NA	10–15	33.1	40.4	5.4/11.6	40.4/NA	No	No	Yes	Postnatally, the newborn’s immunosuppressive therapy was changed to Everolimus with a target level of 5–15 ng/ml. The tumor size continued to decrease significantly. Clinical development remained intact during the first year of life, both cardiac and neurological.
Gonçalves et al. (37)/2025	Giant rhabdomyoma, cardiac dysfunction and pericardial effusion	NA	Single/39 × 35 × 45 mm/LV	SRL/4 mg/day, increased by 1 mg every 3–4 days, up to 8 mg/day	5–10	NA	NA	NA /NA	38.2/C-section	Maternal hypertriglyceridemia	No	Yes	An apical tumor of both ventricles measuring 30 × 15 mm was observed, with no evidence of flow obstruction.

mTORi, mammalian target of rapamycin inhibitor; TSC, tuberous sclerosis complex; FCR, fetal cardiac rhabdomyoma; GA, gestational age; S. E, side effects; FGR, fetal growth restriction; SVT, supraventricular tachycardia; LVOT, left ventricular outflow tract; RVOT, right ventricular outflow tract; RA, right atrium; TV, tricuspid valve; IVS, interventricular septum; LV, left ventricle; RV, right ventricle; SRL, Sirolimus; EVR, Everolimus; EEG, electroencephalogram; NA, not available.

SRL was the most frequently used mTORi drug in 13 pregnancies (86.6%), while EVR was used in 2 cases (13.3%). The most common reason for treatment was progressive tumor growth causing outflow tract obstruction and/or hemodynamic issues such as reduced cardiac output, arrhythmia, and fetal hydrops. The median gestational age at the start of treatment was 30.0 weeks (IQR 26.7–33.1), and the median gestational age at the end of treatment was 38.0 weeks (IQR 36–39). Concerning pregnancy outcomes, 3 were delivered vaginally (20.0%), 6 via cesarean section (40.0%), and in 6 cases the delivery mode was not reported (40.0%).

During treatment, regular monitoring of maternal paraclinical studies and serum mTORi levels was maintained every 1–2 weeks. The dose was gradually adjusted to serum levels, typically kept between 10 and 15 ng/mL. Regarding adverse effects attributed to mTORi, two cases (13.3%) of maternal aphthous ulceration (32, 35), one case (6.6%) of maternal hypertriglyceridemia (37), and one case (6.6%) of fetal growth restriction (6) were reported. All studies documented a reduction in tumor size and improvement in cardiac function during fetal life; however, in five cases (33.3%), tumor regrowth occurred after discontinuing mTORi (26, 27, 29, 32, 35), leading to postnatal reinitiation of therapy. No fetal or neonatal deaths were reported, and none of the 15 cases required postnatal cardiac surgery at hospital discharge.

### Proposed echocardiographic criteria for prenatal administration of mTORi

Based on the evidence gathered from this narrative literature review, we developed the following proposal for echocardiographic criteria to guide the prenatal administration of mTORi. Fetal cardiac rhabdomyomas considered suitable for prenatal therapy are those meeting one or more of the criteria outlined in Table 2.

**Table 2:** Proposed echocardiographic criteria to identify fetuses with FCR eligible for prenatal mTORi therapy (57).

Echocardiographic criterion	Definition
Cardiac inflow obstruction	Tumor causing an altered ventricular filling pattern (monophasic or absent E/A wave) associated with a reduction in cardiac output for the corresponding gestational age
Cardiac outflow tract obstruction	Complete tumor-related obstruction preventing blood flow through the ventricular outflow tract and/or partial obstruction associated with reduced cardiac output for the corresponding gestational age, directed either toward the pulmonary artery or the aorta
Severe atrioventricular valve insufficiency	Regurgitation documented by a complete pulsed Doppler spectrum that reaches the atrial roof on color Doppler evaluation
Fetal rhythm abnormalities	*Supraventricular or ventricular fetal tachycardia*: Fetal heart rate greater than 180 bpm, with an abnormal conduction mechanism documented on rhythm analysis *Complete AV block*: Fetal bradycardia with complete dissociation of atrial and ventricular contractions
Impaired cardiac function	Cardiovascular Profile Score (CVPS) less than 7, with dysfunction conditioned by the FCR
Fetal hydrops	Presence of at least two of the following ultrasound findings attributable to a FCR: pericardial effusion, pleural effusion, ascites, or generalized skin edema >5 mm

*Cardiac Output (CO) = Stroke Volume (SV) × Heart Rate (HR).

*Stroke Volume (SV) = Velocity-Time Integral (VTI) × Valve Area.

*Valve Area = *π* × (diameter /2)^2^.

FCR, fetal cardiac rhabdomyomas; AV, atrioventricular.

### Fetal echocardiographic criteria

#### Cardiac inflow obstruction

Tumor causing an altered ventricular filling pattern (monophasic or absent E/A wave) associated with a reduction in cardiac output for the corresponding gestational age (38, 39).

#### Cardiac outflow tract obstruction

Complete tumor-related obstruction preventing blood flow through the ventricular outflow tract and/or partial obstruction associated with reduced cardiac output for the corresponding gestational age, directed either toward the pulmonary artery or the aorta (40).

#### Severe atrioventricular valve insufficiency

Regurgitation is documented by a complete pulsed Doppler spectrum that extends to the atrial roof on color Doppler evaluation (40).

#### Fetal rhythm abnormalities

##### Supraventricular or ventricular fetal tachycardia

Fetal heart rate greater than 180 bpm, with an abnormal conduction mechanism documented on rhythm analysis.

##### Complete AV block

Fetal bradycardia with complete dissociation of atrial and ventricular contractions (41).

#### Impaired cardiac function

Cardiovascular Profile Score (CVPS) less than 7, with dysfunction conditioned by the FCR (42).

#### Fetal hydrops

Presence of at least two of the following ultrasound findings attributable to an FCR: pericardial effusion, pleural effusion, ascites, or generalized skin edema >5 mm (43, 44).

### Proposed algorithm for the prenatal prescription of mTORi in FCR

**Figure 1:**
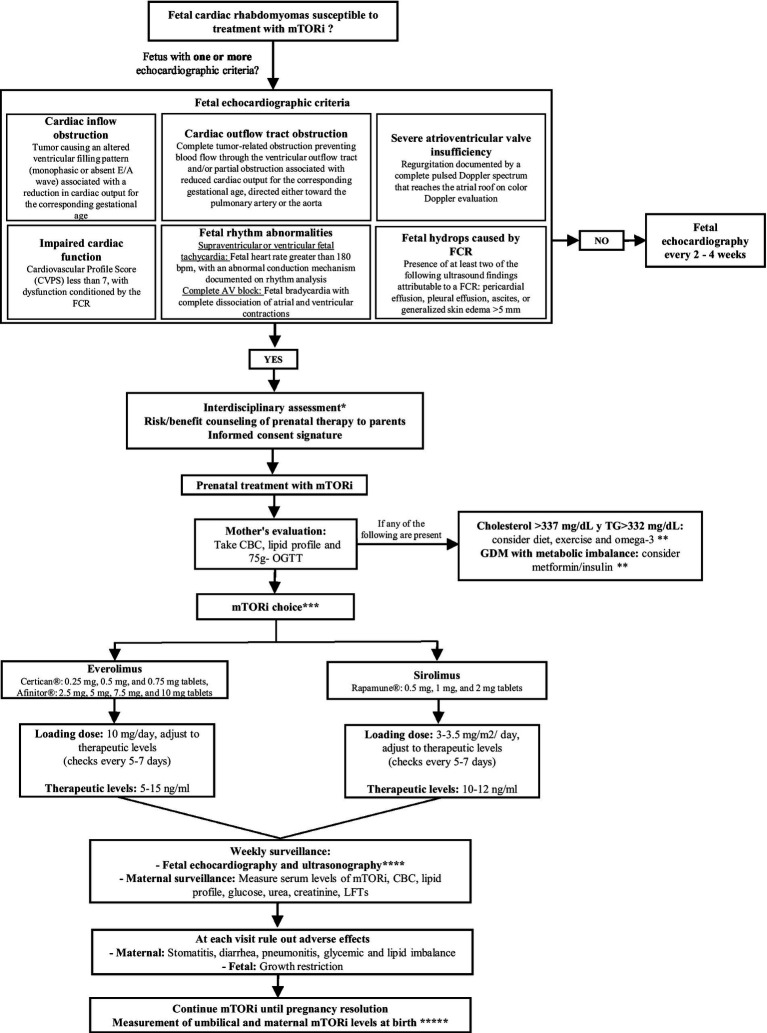
Proposed algorithm for the prenatal administration of mTORi in FCR. mTORi, mTOR inhibitors; FCR, fetal cardiac rhabdomyomas; CBC, complete blood count; OGTT, oral glucose tolerance test; TG, triglycerides; GDM, gestational diabetes mellitus; LFTs, liver function tests. *Interdisciplinary assessment by fetal cardiology, maternal-fetal medicine, perinatal genetics, neuropediatricians and neonatologists. Consider performing neurosonography or fetal magnetic resonance imaging, and prenatal genetic testing for tuberous sclerosis complex. **Safety management of metabolic adverse effects: In the event of dysglycemia or dyslipidemia, management should be directed by an endocrinologist; if abnormalities are clinically significant or persistent despite therapy, temporary interruption or discontinuation of the drug should be considered. ***The choice of the mTORi drug will depend on its availability and the possibility of measuring serum levels for adjustment. ****Echocardiographic monitoring of fetal cardiac function and ultrasonographic monitoring of fetal growth curve and fetal hemodynamics. *****Time of pregnancy resolution according to fetal clinical evolution and obstetric conditions.

Based on the information obtained, an algorithm was developed for the prenatal administration of mTORi in FCR, considered suitable for therapy. The algorithm outlines the steps to follow once the diagnosis is confirmed, the appropriate timing for considering mTORi therapy, as well as recommendations for monitoring and the potential maternal and fetal adverse events associated with this treatment (Figure 1).

## Discussion

### Summary of the main findings

This narrative literature review summarizes 15 reported cases of fetuses with CR, of which 53.3% had a prenatal diagnosis of TSC. SRL was used in 86.6% of cases, while EVR was administered in 13.3%. The most common indication for treatment was progressive tumor growth, leading to outflow tract obstruction and/or hemodynamic compromise, including reduced cardiac output, arrhythmias, and fetal hydrops. Reported adverse events linked to prenatal mTORi therapy included two cases (13.3%) of maternal aphthous ulceration (32, 35), one case (6.6%) of maternal hypertriglyceridemia (37), and one case (6.6%) of fetal growth restriction (6). All studies recorded tumor size reduction and improved fetal cardiac function; however, tumor regrowth was reported in five cases (33.3%) after stopping mTORi (26, 27, 29, 32, 35), leading to postnatal restart of therapy. No fetal or neonatal deaths were reported, and none of the 15 cases required postnatal cardiac surgery at hospital discharge.

### Interpretation of findings and clinical implications

FCR are hamartomatous tumors considered benign; however, their clinical presentation can range from cases without hemodynamic repercussions to severe conditions, including sudden cardiac death, depending on the size, number, and location of the tumors. While small lesions generally do not cause physiological dysfunction and tend to regress spontaneously after birth (13), larger and multiple tumors may lead to outflow tract obstruction, ventricular dysfunction, and arrhythmias, resulting in severe heart failure, hydrops, and fetal death (45). A recent study reported spontaneous tumor regression in 16% of cases before birth and 51% after birth (2), which supports the current recommendation of a conservative approach whenever possible, thereby avoiding the morbidity associated with surgery (1). However, it should be noted that there is up to a 12% risk of fetal death, secondary to cardiac complications caused by rhabdomyomas (2).

In recent years, prenatal treatment with mTORi has been used more often, showing regression of rhabdomyomas in utero (46). In this narrative review of the literature, all studies where prenatal mTORi therapy was given for FCR reported tumor size reduction and improved cardiac function during fetal life, aligning with findings in the international literature (2, 46). However, as noted in this review, there is significant variation in the dosage and treatment protocols for prenatal mTORi therapy. Most of the available evidence comes from case reports or small series, mainly extrapolated from maternal use in kidney transplant recipients, fetal conditions other than FCR, or cases of more severe fetal compromise (13), and no consensus currently exists.

Postnatal treatment with mTORi in children with symptomatic CR and TSC has been shown to cause only mild adverse effects, including dyslipidemia, transient lymphopenia, oral ulcers, alterations in phosphate levels, diarrhea, and constipation. Therefore, postnatal use of mTORi is currently considered a temporary and safe therapeutic option (19, 20). Prenatally, available data on the use of mTORi during the first and second trimesters of pregnancy have not demonstrated congenital malformations (21). In this narrative review, as mentioned above, two cases (13.3%) of maternal aphthous ulceration (32, 35), one case (6.6%) of maternal hypertriglyceridemia (37), and one case (6.6%) of fetal growth restriction (6) were identified. All these potential complications should be discussed with the family before initiating treatment.

### Proposal of echocardiographic criteria to classify FCR susceptible to prenatal treatment with mTORi

Cardiac complications caused by rhabdomyomas in newborns can be life-threatening; therefore, prenatal reduction of tumor burden represents a significant achievement that may prevent adverse perinatal outcomes. Based on the information gathered from this narrative literature review, we propose echocardiographic criteria to classify FCR as susceptible to prenatal therapy with mTORi. The proposal of these echocardiographic criteria is based on the fact that they represent the most frequent complications secondary to FCR, which may lead to a fatal prognosis for the fetus (47) and significant neonatal morbidity (48) (criteria listed in Table 2). Likewise, as demonstrated in the reviewed literature, these criteria encompass the main indications reported in the analyzed case reports for the prenatal administration of mTORi. One of the criteria linked to fetal complications is tumor size, with reports showing that tumors ≥20 mm are significantly connected to neonatal morbidity (48), and tumors ≥30 mm are associated with postnatal arrhythmias requiring treatment (49). However, we did not include tumor size as a criterion for classifying fetuses eligible for prenatal mTORi therapy, since all FCR tend to grow progressively as gestational age increases. In cases of large tumor size, one or more of the proposed criteria for starting prenatal therapy would almost certainly be met.

### Proposed algorithm for prescribing mTORi prenatally in FCR

This review introduces a proposed algorithm for the prenatal use of mTORi in FCR. Its development was based on information from the literature review and other relevant data. The algorithm recommends checking whether any of the proposed echocardiographic criteria are met to identify fetuses that might benefit from prenatal mTORi therapy. If one or more criteria are present, a multidisciplinary evaluation by fetal cardiology and maternal–fetal medicine specialists is advised to determine if additional studies, such as fetal neurosonography or fetal MRI, are needed. This interdisciplinary assessment mandatorily includes pediatric neurology to address long-term neurologic implications; neonatology is also engaged when preterm delivery is anticipated for any reason. Consultation with perinatal genetics is also recommended, as the presence of multiple FCR is considered the earliest clinical biomarker of TSC, occurring in 50–90% of cases (50). Although mutations in TSC1 or TSC2 are major diagnostic criteria for TSC, the difficulty in identifying these variants highlights the importance of imaging studies, since cardiac and brain abnormalities are often the only early signs of TSC (51). Finally, parental counseling should be conducted, explaining the potential benefits and risks of prenatal mTORi therapy, and obtaining informed consent before starting treatment.

The choice of mTORi depends on its availability and the ability to measure serum levels for dose adjustment. For the initial dose of SRL and the target serum level, we followed the recommendations provided by Ebrahimi-Fakhari et al. (31), as this study includes the largest number of reported cases using this therapy for FCR, with target levels comparable to those observed in adult transplant recipients (52). The initial dose of EVR was determined based on recommendations from case reports in the literature (10 mg once daily), the FDA-approved dose for breast cancer, neuroendocrine tumors, renal cell carcinoma, and renal angiomyolipomas (29, 34). Maternal target serum levels of SRL and EVR, ranging from 10 to 15 ng/mL, are based on guidelines for the use of mTORi in adult transplant recipients and are the most frequently referenced in the reviewed literature (52). The dose of everolimus used for prophylaxis against solid organ transplant rejection is lower (0.75 mg twice daily), and therefore the serum level of everolimus used for monitoring is 3–8 ng/mL (18). Maternal serum mTORi levels are recommended to be monitored weekly, since a direct relationship has been shown between the pharmacokinetics of SRL in maternal and fetal serum levels. This suggests that fetal SRL dosage can be adjusted by measuring maternal serum concentrations, making it feasible to monitor maternal blood every 5 to 7 days until the target range is achieved (53). Maternal and fetal monitoring focuses on the main adverse events reported secondary to the administration of mTORi. Maternal complications include bone marrow suppression, disruption of mucosal barriers, impaired wound healing, and changes in lipid and carbohydrate metabolism, while the primary fetal complication reported is growth restriction (2, 15, 54).

A recently published systematic review by Muschel et al. (55) provides case-based evidence on the prenatal use of mTOR inhibitors. The fetal indications reported align with our proposed echocardiographic criteria for identifying FCR eligible for prenatal mTORi therapy and indicate clear fetal benefit. Nonetheless, treatment may require discontinuation because of unfavorable maternal effects—such as drug-induced cough and diabetogenic changes—described in their report. These observations underscore the need for comprehensive pre-treatment counseling and vigilant maternal monitoring for adverse effects, both of which we have incorporated as key components of our algorithm.

### Strengths and limitations of the study

The main strengths of this study lie in the information obtained from a narrative literature review conducted using major international databases, making it one of the largest reviews of case reports and case series of FCR to date, and including data on two different mTOR inhibitors. This provides an overview of the approaches implemented for the prenatal use of mTORi, as well as valuable information on the potential adverse effects associated with this therapy. Data extraction was performed independently by two reviewers, which minimized the risk of interpretation bias and errors in variable coding. Discrepancies were resolved by consensus, thereby strengthening the reliability of the qualitative analysis of the included cases. This process enhanced the internal validity of the reported results. The proposed criteria to classify fetuses as susceptible to prenatal treatment with mTORi, along with the management algorithm, offer a contrast to the current international reality. Using the information generated by this study may help facilitate and promote the adoption of prenatal therapy with mTORi in a more standardized and comprehensive manner. However, we acknowledge as limitations of this narrative literature review the small number of studies, all of which had a retrospective and non-randomized design, with heterogeneity in prenatal mTORi therapy and varying follow-up periods. Only a few studies included information on long-term outcomes, each reporting different aspects. Publication bias and the methodological quality of the studies were not assessed. The publication years of the included studies span the last 25 years, during which significant advances have occurred in prenatal diagnosis and perinatal management compared with earlier cases. Additionally, most reported cases were predominantly from the third trimester, which limits the generalizability of the findings. The proposed criteria to classify fetuses as susceptible to prenatal mTORi therapy, along with the management algorithm, were developed using the limited information currently available and have not been prospectively validated. Therefore, these criteria are likely to be refined as better-designed studies and additional case reports become available.

### Future research

It is essential to conduct multicenter studies and randomized clinical trials to assess the efficacy and safety of mTORi for prenatal treatment of FCR. Additionally, the long-term effects on newborns exposed to these drugs during gestation must be examined. Gathering more data on the clinical progression of these patients will help develop stronger, evidence-based treatment guidelines. A promising project is KaRhab, an international online registry for cardiac rhabdomyomas, which is expected to provide valuable information (56).

## Conclusion

Prenatal treatment with mTORi has demonstrated potential benefits in specific cases of FCR with hemodynamic compromise. In the absence of international consensus, this study proposes echocardiographic criteria and a management algorithm for their use, which require validation in future research. Clinical trials are necessary to determine optimal dosing, maternal–fetal monitoring, and long-term outcomes more accurately. Incorporating this therapy into clinical practice, supported by solid evidence, could significantly improve the prognosis of fetuses affected by this condition.
