# Supplementary material for: Fetal cardiac rhabdomyomas susceptible to prenatal treatment with mTOR inhibitors: literature review and proposal of a prenatal management algorithm
Source: Front Med (Lausanne). 2025 Dec 8;12:1711774. doi: 10.3389/fmed.2025.1711774 (PMC12719440; doi:10.3389/fmed.2025.1711774)
Supplement: Supplementary file 1 [file Table_1.docx]

Supplementary Material

**Supplementary Table 1.** Literature search strategy by databases conducted on 28/02/2025, including articles published from 01/01/2000 to 28/02/2025

| **Set** | **PubMed** | **Results** |
| --- | --- | --- |
| ("Rhabdomyoma"[MeSH Terms] OR "cardiac rhabdomyoma"[tiab] OR "fetal rhabdomyoma"[tiab]) AND ("Sirolimus"[MeSH Terms] OR "Everolimus"[MeSH Terms] OR "mTOR inhibitor"[tiab] OR "Sirolimus"[tiab] OR "Everolimus"[tiab]) AND ("Prenatal"[tiab] OR "transplacental therapy"[tiab]) | | |
| Filters applied:  Publication date: From 2000/1/1 - 2025/02/28, Article language: English or Spanish, Species: Humans | | |
| #1 | ("Rhabdomyoma"[MeSH Terms] OR "cardiac rhabdomyoma"[tiab] OR "fetal rhabdomyoma"[tiab]) | **654** |
| #2 | AND ("Sirolimus"[MeSH Terms] OR "Everolimus"[MeSH Terms] OR "mTOR inhibitor"[tiab] OR "Sirolimus"[tiab] OR "Everolimus"[tiab]) | **60** |
| #3 | AND ("Prenatal"[tiab] OR "transplacental therapy"[tiab]) | **12** |
|  | Selected articles | **5** |
| Set | **Scopus** | Results |
| TITLE-ABS-KEY("cardiac rhabdomyoma" OR "fetal rhabdomyoma" OR "rhabdomyoma") AND TITLE-ABS-KEY("sirolimus" OR "everolimus" OR "mTOR inhibitor") AND TITLE-ABS-KEY("prenatal" OR "transplacental therapy") | | |
| Filters applied:  Year range: 2000 – 2025, Subject area: Medicine, Document type: Article, Language: English or Spanish | | |
| #1 | TITLE-ABS-KEY("cardiac rhabdomyoma" OR "fetal rhabdomyoma" OR "rhabdomyoma") | **408** |
| #2 | AND TITLE-ABS-KEY("sirolimus" OR "everolimus" OR "mTOR inhibitor | **83** |
| #3 | AND TITLE-ABS-KEY("prenatal" OR "transplacental therapy") | **21** |
|  | Selected articles | **5** |
| Set | **Web of Science** | Results |
| TS=("cardiac rhabdomyoma" OR "fetal rhabdomyoma" OR "rhabdomyoma") AND TS=("sirolimus" OR "everolimus" OR "mTOR inhibitor") AND TS=("prenatal" OR "transplacental therapy") | | |
| Filters applied:  Year range: 2000.01.01 –2025.02.28, Document type: Article, Language: English or Spanish | | |
| #1 | TS=("cardiac rhabdomyoma" OR "fetal rhabdomyoma" OR "rhabdomyoma") | **166** |
| #2 | AND TS=("sirolimus" OR "everolimus" OR "mTOR inhibitor") | **37** |
| #3 | AND TS=("prenatal" OR "transplacental therapy") | **13** |
|  | Selected articles | **5** |
| Set | **Google Scholar** | Results |
| ("fetal rhabdomyoma" OR "cardiac rhabdomyoma") AND ("mTOR inhibitor" OR "sirolimus" OR "everolimus") AND ("prenatal treatment" OR "transplacental therapy") | | |
| Filters applied:  Year range: 2000 – 2025, Document type: Any type, Language: Any language | | |
| #1 | ("fetal rhabdomyoma" OR "cardiac rhabdomyoma") | **5,310** |
| #2 | AND ("mTOR inhibitor" OR "sirolimus" OR "everolimus") | **1,210** |
| #3 | AND ("prenatal treatment" OR "transplacental therapy") | **50** |
|  | Selected articles | **12** |
