# Supplementary material for: Fetal cardiac rhabdomyomas susceptible to prenatal treatment with mTOR inhibitors: literature review and proposal of a prenatal management algorithm
Source: Front Med (Lausanne). 2025 Dec 8;12:1711774. doi: 10.3389/fmed.2025.1711774 (PMC12719440; doi:10.3389/fmed.2025.1711774)
Supplement: Supplementary file 2 [file Table_2.docx]

Supplementary Material

**Supplementary Table 2.** Articles selected from the literature search by databases

| **Set** | **PubMed** | **DOI** |
| --- | --- | --- |
| Ilina D Pluym, 2020 | Fetal cardiac rhabdomyomas treated with maternal sirolimus | **10.1002/pd.5613** |
| Daniel Ebrahimi-Fakhari, 2021 | Prenatal Sirolimus Treatment for Rhabdomyomas in Tuberous Sclerosis | **10.1016/j.pediatrneurol.2021.09.014** |
| Sergio Cavalheiro, 2021 | Everolimus as a possible prenatal treatment of in utero diagnosed subependymal lesions in tuberous sclerosis complex: a case report | **10.1007/s00381-021-05218-4** |
| Ana Dagge, 2022 | Fetal Tuberous Sclerosis: Sirolimus for the Treatment of Fetal rhabdomyoma | **10.1080/15513815.2021.1948646** |
| J. Griesman, 2024 | Transplacental therapy with sirolimus for non-tuberous sclerosis rhabdomyoma in fetus | **10.1002/uog.29103** |
| **Set** | **Scopus** | **DOI** |
| Ilina D Pluym, 2020 | Fetal cardiac rhabdomyomas treated with maternal sirolimus | **10.1002/pd.5613** |
| Sergio Cavalheiro, 2021 | Everolimus as a possible prenatal treatment of in utero diagnosed subependymal lesions in tuberous sclerosis complex: a case report | **10.1007/s00381-021-05218-4** |
| Daniel Ebrahimi-Fakhari, 2021 | Prenatal Sirolimus Treatment for Rhabdomyomas in Tuberous Sclerosis | **10.1016/j.pediatrneurol.2021.09.014** |
| Ana Dagge, 2022 | Fetal Tuberous Sclerosis: Sirolimus for the Treatment of Fetal rhabdomyoma | **10.1080/15513815.2021.1948646** |
| Patrick Schenk, 2024 | Prenatal initiation of therapy with mTOR inhibitors for giant cardiac rhabdomyoma | **10.1055/a-2408-1068** |
| **Set** | **Web of Science** | **DOI** |
| Daniel Ebrahimi-Fakhari, 2021 | Prenatal Sirolimus Treatment for Rhabdomyomas in Tuberous Sclerosis | **10.1016/j.pediatrneurol.2021.09.014** |
| Sergio Cavalheiro, 2021 | Everolimus as a possible prenatal treatment of in utero diagnosed subependymal lesions in tuberous sclerosis complex: a case report | **10.1007/s00381-021-05218-4** |
| Ana Dagge, 2022 | Fetal Tuberous Sclerosis: Sirolimus for the Treatment of Fetal rhabdomyoma | **10.1080/15513815.2021.1948646** |
| Will, Joachim Carsten, 2023 | Successful Prenatal Treatment of Cardiac Rhabdomyoma in a Fetus with Tuberous Sclerosis | **10.3390/pediatric15010020** |
| Gonçalves, 2025 | Transplacental sirolimus for reversal of fetal heart failure due to fetal cardiac rhabdomyoma: fetal and maternal considerations | **10.1159/000542664** |
| **Set** | **Google Scholar** | **DOI** |
| Barnes, Benjamin T, 2018 | Maternal sirolimus therapy for fetal cardiac rhabdomyomas | **10.1056/NEJMc1800352** |
| Hyea Park, 2019 | Sirolimus therapy for fetal cardiac rhabdomyoma in a pregnant woman with tuberous sclerosis | **10.5468/ogs.2019.62.4.280** |
| Vachon-Marceau C, 2019 | In-utero treatment of large symptomatic rhabdomyoma with sirolimus | **10.1002/uog.20196** |
| Daniel Ebrahimi-Fakhari, 2021 | Prenatal Sirolimus Treatment for Rhabdomyomas in Tuberous Sclerosis | **10.1016/j.pediatrneurol.2021.09.014** |
| Sergio Cavalheiro, 2021 | Everolimus as a possible prenatal treatment of in utero diagnosed subependymal lesions in tuberous sclerosis complex: a case report | **10.1007/s00381-021-05218-4** |
| Ana Dagge, 2021 | Fetal Tuberous Sclerosis: Sirolimus for the Treatment of Fetal rhabdomyoma | **10.1080/15513815.2021.1948646** |
| Anita Maász, 2023 | Three-Year Follow-Up after Intrauterine mTOR Inhibitor Administration for Fetus with TSC-Associated Rhabdomyoma | **10.3390/ijms241612886** |
| Will, Joachim Carsten, 2023 | Successful Prenatal Treatment of Cardiac Rhabdomyoma in a Fetus with Tuberous Sclerosis | **10.3390/pediatric15010020** |
| McLoughlin, 2023 | Massive fetal cardiac rhabdomyoma treated with transplacental sirolimus | **10.5114/pcard.2022.127365** |
| J. Griesman, 2024 | Transplacental therapy with sirolimus for non-tuberous sclerosis rhabdomyoma in fetus | **10.1002/uog.29103** |
| Patrick Schenk, 2024 | Prenatal initiation of therapy with mTOR inhibitors for giant cardiac rhabdomyoma | **10.1055/a-2408-1068** |
| Gonçalves, 2025 | Transplacental sirolimus for reversal of fetal heart failure due to fetal cardiac rhabdomyoma: fetal and maternal considerations | **10.1159/000542664** |
